# Resveratrol as an Adjunct Antiviral Agent in Pediatric Viral Infections: A Review on Mechanistic Insights and Gut Microbiota Modulation

**DOI:** 10.3390/ijms262311341

**Published:** 2025-11-24

**Authors:** Roberta Leonardi, Manuela Lo Bianco, Salvatore Spinello, Pasqua Betta, Caterina Gagliano, Vittorio Calabrese, Agata Polizzi, Giulia Malaguarnera

**Affiliations:** 1 Postgraduate Training Programme in Pediatrics, Department of Clinical and Experimental Medicine, University of Catania, 95123 Catania, Italy; leonardi.roberta@outlook.it; 2 Neonatal Intensive Care Unit, AOU Policlinico G. Rodolico San Marco, 95123 Catania, Italy; mlbetta@yahoo.it; 3 Unit of Pediatric Clinic, Department of Clinical and Experimental Medicine, University of Catania, 95123 Catania, Italy; lobianco.manuela@gmail.com (M.L.B.); agata.polizzi1@unict.it (A.P.); 4 Federazione Italiana Medici di Medicina Generale-F.I.M.M.G. (Italian Federation of General Practitioners), 00153 Rome, Italy; salvospinello@gmail.com; 5 Department of Medicine and Surgery, University of Enna “Kore”, Piazza dell’Università, 94100 Enna, Italy; caterina.gagliano@unikore.it; 6 Eye Center G.B. Morgagni-DSV, 95125 Catania, Italy; 7 Department of Biomedical and Biotechnological Sciences, University of Catania, 95123 Catania, Italy; calabres@unict.it; 8 Department of Human Sciences and Quality of Life Promotion, San Raffaele Roma Open University, 00166 Rome, Italy

**Keywords:** resveratrol, pediatric viral infections, gut microbiota, antiviral mechanisms, dysbiosis

## Abstract

Pediatric viral infections impose a heavy burden on child health, often worsened by infection-induced gut dysbiosis. Resveratrol, a natural polyphenol with antiviral, anti-inflammatory, and microbiota-modulating properties, has been proposed to interrupt this pathogenic feedback. To our knowledge, this is the first narrative review focused on resveratrol’s antiviral activity in pediatric viral infections, concurrently evaluating its impact on the gut microbiota and their interrelationship. We synthetized preclinical and the limited available pediatric clinical data regarding resveratrol’s effect on SARS-CoV-2, respiratory syncytial virus, influenza, rotavirus, and norovirus, extracting information on the models, routes of administration, dosages, mechanisms, and outcomes. Resveratrol interferes with viral lifecycles via diverse mechanisms (modulation of host signaling cascades, capsid or structural protein interactions, and suppression of pro-viral chaperones) while concurrently reshaping the gut microbiota (reducing opportunistic taxa and enriching beneficial genera such as *Bifidobacterium* and *Lactobacillus*) leading to improved short-chain fatty acid profiles, barrier integrity, and dampened inflammation. Intranasal resveratrol in children shows clinical benefit, while oral use is underexplored and limited by poor bioavailability; adult data hint at supportive microbiome and anti-inflammatory effects if the delivery is optimized. These dual antiviral and microbiome-directed effects position resveratrol as a promising adjunct in pediatric viral disease management, though well-powered pediatric clinical trials are needed to define dosages, delivery strategies, and the contribution of microbiota-mediated synergy.

## 1. Introduction

Pediatric viral infections pose a major public health challenge, contributing significantly to morbidity and mortality [1]. Among these, respiratory syncytial virus (RSV) stands out as a leading cause of severe respiratory disease in young children, particularly in tropical regions where its impact on childhood mortality may be underestimated [2]. RSV is responsible for a substantial proportion of acute lower respiratory infections (ALRI) in children, leading to high hospitalization rates and significant global mortality [3]. The disease burden is especially pronounced in infants under two years of age, with nearly all children experiencing at least one RSV infection by this time [4].

Moreover, the severity of viral infections in children is further influenced by co-infections, which can complicate diagnosis, treatment, and clinical outcomes [5,6]. Evidence suggests that multiple viral pathogens frequently coexist in pediatric respiratory infections, with RSV often playing a key role in determining disease severity [6]. In particular, co-infections involving RSV and other respiratory viruses, such as rhinovirus and human bocavirus, have been associated with extended hospitalization and worsened morbidity [7,8].

The emergence of novel viral pathogens, such as SARS-CoV-2, has further complicated the landscape of pediatric viral infections. Children infected with SARS-CoV-2 display a wide spectrum of clinical presentations, and concurrent infections with other respiratory viruses have been linked to more severe disease outcomes [9,10]. During the COVID-19 pandemic, children up to 12 years continued to show a high circulation of respiratory viruses, with enterovirus/rhinovirus (EV/RV) and respiratory syncytial virus (HRSV) being the most prevalent. As reported by Dallmeyer et al. [11], infants aged 0–1 year were the most affected, and HRSV showed a marked resurgence following the relaxation of containment measures. Among the identified viruses, enteroviruses, adenoviruses, and human bocavirus are also well recognized to cause gastrointestinal symptoms such as diarrhea, vomiting, and abdominal pain. This highlights the clinical relevance of monitoring both respiratory and digestive manifestations in pediatric viral infections [11]. Additionally, asymptomatic children with high viral loads raise concerns regarding their role in community transmission [12,13].

Beyond respiratory pathogens, viral gastroenteritis remains a significant threat in pediatric populations, with rotavirus and norovirus being the primary etiological agents [14,15]. Despite the availability of vaccines, insufficient immunization coverage continues to contribute to disease-related morbidity [16,17]. The highest burden of viral gastroenteritis occurs in children under five years of age, resulting in considerable hospitalization rates and increased healthcare costs [16].

The impact of viral infections in children is shaped by multiple factors, including the type of pathogen, co-infections, and the child’s overall health status, including gut microbiota modifications.

Pediatric viral infections and gut microbiota alterations are bidirectionally linked, as infections can disrupt microbial composition, while dysbiosis may compromise immune responses, increasing susceptibility to further infections, particularly in children [18]. Regarding the importance of gut microbiota in pediatric viral infections, hospitalized children with acute gastroenteritis displayed distinct microbial profiles, influenced by viral pathogens such as rotavirus and norovirus, in conjunction with bacterial agents like enteropathogenic *Escherichia coli* [18].

Although respiratory viruses such as influenza and RSV are primarily respiratory illnesses, they can cause gastrointestinal manifestations or be responsible for changes in the gut microbiota, increasing the disease’s severity and predisposing children to secondary bacterial infections [19,20,21]. In addition, during the COVID-19 pandemic, several studies indicated that gut microbiota dysbiosis correlates with inflammatory markers, potentially influencing immune responses and disease severity [19].

Among various prebiotics with antiviral potential through gut microbiota modulation, resveratrol has been investigated in several studies for its efficacy in influencing viral infections.

Resveratrol (trans-3,4,5-trihydroxystilbene) is a stilbene, belonging to the class of polyphenols, which are natural compounds found in rhubarb, berries, nuts, and red grapes [22]. Resveratrol is the most studied stilbenoid because of its numerous biological properties: it has anticancer, neuroprotective, cardioprotective, and of particular relevance to our topic, antiviral, antioxidative, immune-regulatory, and anti-inflammatory properties [23,24,25,26]. In particular, resveratrol showed inhibitory activity against several viruses [27], including herpes simplex [28], varicella-zoster virus (VZV) [29], influenza A [30], MERS-CoV, and SARS (Severe Acute Respiratory Syndrome) [31]. Taking into consideration that resveratrol is metabolized by gut microbiota [32] and that, in turn, resveratrol can influence gut bacterial diversity [33], this bilateral relationship between the two could represent a crucial element in the treatment’s efficiency [34]. Additionally, features of probiotic bacteria, notably biofilm formation and adhesion to enterocytes, can be enhanced by the association with resveratrol [35,36].

In this review we aim to describe the molecular mechanism of action and the possible therapeutic use of resveratrol in some infantile viral infections, highlighting the association between resveratrol and gut microbiota.

## 2. Outline of the Review

The manuscript is structured as follows. First, we summarize the bidirectional relationships between pediatric viral infections and the gut microbiota, emphasizing pathogen-specific dysbiotic signatures and their clinical relevance. We then examine evidence that resveratrol modulates intestinal communities and metabolite profiles, and consider mechanistic links through which these microbiome changes could enhance antiviral host defenses. The main body synthesizes preclinical and clinical data on resveratrol’s direct antiviral actions, with focused subsections on RSV, influenza, rotavirus, and norovirus. This is followed by a critical appraisal of pediatric clinical experience, administration routes, and formulation challenges, and a discussion of safety, dosing considerations, and potential combinatory strategies. Our review concludes with a concise summary of knowledge gaps and prioritized recommendations for translational and clinical research.

## 3. Viral Infections and Gut Microbiota Alterations in Children

While the term “microbiota” refers to the full assemblage of microorganisms (bacteria, archaea, fungi, protozoa, and viruses) that reside in a particular habitat or host, microbiome encompasses the collective genetic material of those microbes. This distinction is pivotal for analyzing and categorizing microbial populations, as illustrated by the Human Microbiome Project [37]. Using next-generation sequencing, the Project surveyed various human body sites, highlighting the gut as harboring the densest and most diverse microbial community, largely composed of the *Firmicutes* and *Bacteroidetes* phyla. Through 16S rRNA gene profiling and foundational metagenomic methods, researchers observed substantial interindividual variation in gut microbial composition, underscoring the importance of these communities in metabolic processes, immune function, and overall health [37].

The gut–lung axis is represented by the mucous immune system consisting of the GI tract and the respiratory tract [38]. The gut and lung microbiota, as a part of the gut–lung axis, can modulate immune responses [39,40,41]. Once the presence of lung bacteria was discovered, it was highlighted that they are mainly composed of *Bacteroidetes* and *Firmicutes*, like gut microbiota, but also by *Proteobacteria* [42,43,44]. Consequently, <<lung microbiota>> dysbiosis, or alterations in saprophytes composition, such as as primum movens of opportunistic pathogenic colonizations, causes respiratory infections [43,44].

Recently, it has been shown that gut microbiota has a fundamental function in modeling respiratory inflammation [45]. Gut microbiome and respiratory tract infections influence each other, as in the case of influenza virus infection [46]. This is due to a cross-talk between the respiratory tract and GI tract’s microbiome [47]. This bidirectional interaction underscores the necessity of an integrated perspective in understanding pediatric viral infections.

**Figure 1:**
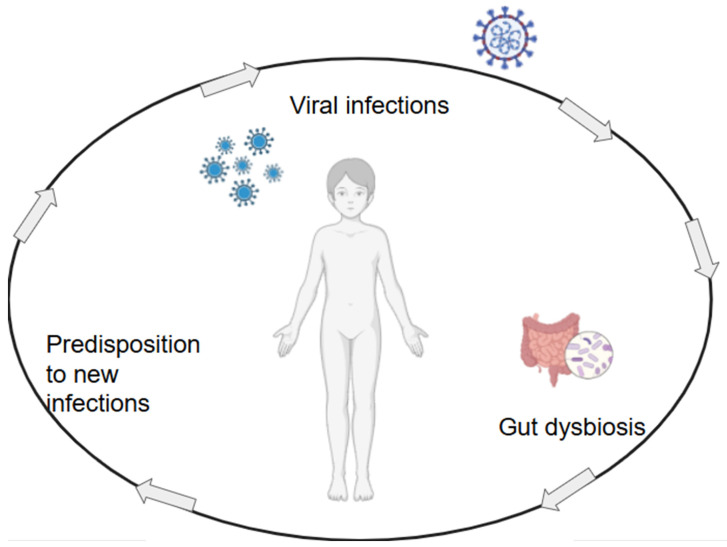
The interplay between viral infections and gut microbiota alterations in children. Figure created with Biorender.com.

The reciprocity of influence between viral infections and microbiota is depicted in Figure 1, and this acts through several mechanisms. Viral infections in children can profoundly alter gut microbiota composition, influencing immune responses and increasing susceptibility to secondary infections. Additionally, because of its impact in infections’ severity, the gut microbiota modulation has been suggested to be a possible parameter to assess the risk of progression of respiratory infections [48]. Influenza virus infection compromises gut barrier integrity, leading to dysbiosis characterized by diminished short-chain fatty acid production, which plays a crucial role in gut homeostasis [21]. Such microbiome disturbances can amplify disease severity and complicate recovery trajectories. The implications of viral infections extend beyond acute gastrointestinal symptoms. For example, norovirus infection has been associated with reduced microbial diversity, a key factor in maintaining resistance against opportunistic pathogens [49].

Fadlyana et al. highlight that dysbiosis can enhance the risk of infections, not only in the gastrointestinal tract, but also in distant organs such as the lungs, due to the immune-modulating effects of gut microbiota metabolites [50]. Furthermore, studies have shown that a diverse gut microbiota is essential for maintaining protective immunity against viral infections. For example, Nelson et al. demonstrated that the disruption of the gut microbiota following norovirus infection can compromise colonization resistance against pathogens, including viruses like norovirus, which can lead to gastroenteritis [49]. The loss of microbial diversity following viral infections can exacerbate the severity of subsequent infections, as seen in children with acute gastroenteritis, who often exhibit reduced gut microbiota diversity compared to healthy controls [18].

The interplay between gut microbiota and the immune system is complex. Liu et al. noted that the gut microbiota can modulate immune responses, potentially enhancing antiviral defenses [51]. This is particularly relevant in the context of respiratory viral infections, where the gut–lung axis plays a significant role in immune regulation. For instance, Altomare et al. emphasized that a healthy gut microbiota can enhance immune responses in the lungs, thereby offering protection against respiratory viral infections [52]. Therefore, maintaining gut health may be crucial for preventing viral infections in children. Moreover, the gut microbiota’s influence extends to the modulation of inflammatory responses during viral infections. In fact, Liu et al. found that dysbiosis following respiratory viral infections could lead to an imbalance in immune homeostasis, increasing susceptibility to secondary infections [53].

Specific gut microbiota alterations due to viral infections in children are summarized in Table 1.

Microbiota alterations in COVID-19 patients are specifically characterized by poor microbes’ heterogeneity associated with enrichment of opportunistic pathogens [61] including *Streptococcus*, *Erysipelatoclostridium*, *Rothia*, *Actinomyces*, and *Veillonella* [62]. Compared to healthy controls, COVID-19 children’s gut microbiota was enriched in opportunistic pathogenic bacteria, such as *Pseudomonas*, *Herbaspirillum and Burkholderia*, with a reduction in typical commensal bacteria [54].

Zuo et al. showed gut microbiota alterations in COVID-19 patients, including the increase in *Clostridium ramosum*, *Coprobacillus*, and *Clostridium hathewayi*, directly proportional to COVID-19 severity, and the reduction in commensal bacteria, such as *Faecalibacterium prausnitzii*, an anti-inflammatory microorganism [19]. Dysbiosis persisted even when throat swabs were negative and symptoms had resolved [19]. In addition, there were inverse correlations between *Bacterioides dorei*, *Bacteroides ovatus*, *Bacteroides thetaiotamicron*, and *Bacteroides massiliensis* that were able to downregulate colonic expression of ACE-2 [63] and SARS-CoV-2 fecal shedding [19].

Fecal samples with high SARS-CoV-2 infectivity were rich in opportunistic pathogens like *Streptococcus infantis*, *Morganella morganii*, and *Collinsella aerofaciens*, while those with low-to-no SARS-CoV-2 infectivity were rich in short-chain fatty acids and tryptophan producers, like *Lachnospiraceae bacterium* [64].

Particularly, the underlying mechanism of virus-induced microbiome dysbiosis depends on enterocytes’ ACE2 expression [38,42]. ACE2 controls the gut tryptophan uptake, and tryptophan plays an important role in synthesis of antimicrobial peptides [42]. Therefore, the loss of ACE2 consequent to SARS-CoV-2 infection, leads to a reduction in antimicrobial peptides, and then to pathogen growth, longevity and gut dysbiosis, but also amplifies gut barrier permeability alterations, and local and systemic immunity inefficiency [65,66,67].

Significant differences in gut microbiota composition between healthy infants and those infected with RSV have been demonstrated, emphasizing its potential role in disease severity. Analysis of stool samples from hospitalized infants with RSV revealed an enrichment of *Clostridiales*, *Odoribacteraceae*, *Lactobacillaceae*, and *Actinomyces*, alongside significant beta diversity alterations compared to healthy controls [20]. Infants with severe RSV disease exhibited lower alpha diversity, suggesting a reduction in microbial richness. While the causal relationship remains unclear, these findings support the hypothesis that gut dysbiosis contributes to RSV severity via the gut–lung axis [55]. Further evidence from bronchiolitis studies suggests that infants with a Bacteroides-dominant gut profile had a higher likelihood of developing severe RSV-related bronchiolitis, compared to those with a Bacteroides-dominant profile [56].

Two recent studies from Li et al. 2019 [57,58] investigated gut microbiota profiles in children with recurrent respiratory tract infections (RRTIs). The first, involving 26 children with RRTIs and 23 healthy controls, reported significantly lower alpha diversity in the RRTI group, with notable shifts in specific phyla and genera (e.g., increased Enterococcus) [57]. The second study, examining 90 children with RRTIs versus 30 controls, highlighted a marked reduction in beneficial bacteria (particularly lactobacilli and bifidobacteria) in infected children [58].

Regarding gastroenteritis and, specifically, norovirus infections, a recent study demonstrated a marked gut dysbiosis with *Veillonella* being the dominant genus in infected children, as well as *Enterococcus faecium*. Instead, beneficial butyrate-producing bacteria such as *Faecalibacterium*, *Blautia*, *Subdoligranulum*, *Eubacterium Hallii group*, *Fusicatenibacter*, *Agathobacter*, *Roseburia*, and *Dorea*, which normally play critical roles in gut homeostasis and anti-inflammatory responses, were significantly depleted [59].

A recent study focused on how rotavirus infection alters the gut microbiota composition of children, examining fecal samples from patients infected with RV, both before and after treatment, alongside healthy controls. It has been shown that RV infection significantly reduces overall microbial diversity and increases Proteobacteria abundance, suggesting a notable shift toward dysbiosis. Moreover, only a small subset of differentially abundant genera displayed partial recovery post-treatment, indicating that the gut microbiota may not readily return to a healthy state in the short term [60].

The gut microbiota dysbiosis–immune hyperresponse–inflammation triad could also explain individual response to drugs and nutraceuticals, in particular because of the interference with bioavailability and pharmacokinetics made by gut microbiota [66].

Taken together, the importance of an action that could regulate gut microbiota and consequently immunity, especially in children, can be suggested. Personalized diet strategies, but also probiotics and prebiotics may be a useful supplement to ordinary therapies [35].

## 4. Effect of Resveratrol on Gut Microbiota Composition

Several articles recently demonstrated the capacity of resveratrol to induce changes in gut microbiota composition. This is due to its direct influence on a healthy microbiota composition or is related to the activity of resveratrol’s byproducts [34]. A study conducted in humans treated with resveratrol for 4 months found alterations in urinary derivatives of amino acids, which reflect the composition of the gut microbiota [68]. These results support the thesis of the direct activity of resveratrol in the modulation of gut microbiota in humans, in accordance with previous studies in rodents [69]. The changes concerned tyrosine-derived, tryptophan-derived, phenylalanine-derived, and histidine-derived metabolites, which are produced and degraded by gut bacteria and then excreted in the urine and which have been related to intestinal dysfunction, blood pressure, and body weight. [68,70]

It is assumed that the health gains of resveratrol in the gut are based on the gut microbiota. For instance, resveratrol reduces the number of opportunistic pathogens in vivo [71]. A study with several rodent models has shown that dietary resveratrol induces selective remodeling of the intestinal microbiota, characterized by a decrease in opportunistic taxa (e.g., *Enterococcus faecalis* and *Escherichia coli*) and a concomitant increase in putatively beneficial genera such as *Lactobacillus* and *Bifidobacterium*. These compositional shifts, observed at dosing regimens ranging from low mg·kg^−1^ daily in DSS-colitis models (e.g., 1 mg·kg^−1^·day^−1^) to higher dietary supplementation over weeks (e.g., 200–400 mg·kg^−1^ for 8–12 weeks), were associated with reduced oxidative stress markers, activation of Nrf2-dependent antioxidant responses, suppression of NF-κB signaling, and, in several studies, improvements in barrier integrity and short-chain fatty acid-related readouts, thereby linking microbiota modulation to the gut-protective effects of resveratrol [71].

Resveratrol supplementation can alter gut microbiota also through its antimicrobial activity, which is effective against both Gram-negative and Gram-positive pathogens [72]. For example, resveratrol has been shown to reduce the abundance of *E. coli* and Enterobacteria in rats, and at the same time it increased *Bifidobacterium* and *Lactobacillus*, restoring a healthy microbiota phenotype [69].

Resveratrol can inhibit the growth of various Clostridia species [73]. Giuliani and colleagues investigated the effects of a dietary supplement containing trans-resveratrol combined with ε-viniferin using the Simulator of the Human Intestinal Microbial Ecosystem, a validated in vitro model that reproduces human colonic conditions. Continuous exposure via the simulator feed for ten days followed by a four-day washout enabled longitudinal monitoring via denaturing gradient gel electrophoresis and 16S ribosomal RNA gene amplicon sequencing, together with metabolic measurements including short-chain fatty acids and ammonium. The intervention induced a proximal colon increase in Enterobacteriaceae concurrent with a decrease in *Bifidobacteriales*; these compositional shifts only partially reverted after washout [74]. These controlled, human-relevant dynamics support a direct microbiota-mediated component to resveratrol’s intestinal effects. 

Firmicutes and Bacteroidetes are the most represented phyla in human microbiome, sometimes comprising more than 90% of the total percentage [75]. Important findings concern the capacity of resveratrol in reducing the F/B ratio [76,77,78], while an enhanced F/B ratio has been related to a higher risk of obesity and other pathologies in human and mice [37,79]. In a recent randomized, double-blind trial, 37 subjects who were overweight and obese received epigallocatechin-3-gallate and resveratrol or placebo for 12 weeks [80]. Although the actual contribution of resveratrol cannot be assessed, having been used together with epigallocatechin-3-gallate, this study demonstrated that this supplementation significantly decreased the abundance of Bacteroidetes (*p* = 0.05), which resulted in a decreased *Bacteroidetes*/*Firmicutes* ratio because of the absence of effects on Firmucutes and also tended to reduce *Faecalibacterium prausnitzii* (*p* = 0.10) as compared with the placebo [80]. The capacity of resveratrol to reduce Bacteroides is important because they can be highly pathogenic, being antibiotic-resistant bacteria, and also because high levels of these bacteria can provoke inflammation [71].

In addition, resveratrol showed prebiotic-like properties by increasing the percentage of *Bifidobacterium* strains [33,76]. For instance, resveratrol has been shown to reshape gut microbiota profiles in colitis models by suppressing pro-inflammatory genera (e.g., *Akkermansia*, *Dorea*, *Sutterella*, and *Bilophila*) and promoting beneficial taxa such as Bifidobacterium in dextran sulfate sodium-treated mice. In db/db mice, resveratrol supplementation also reversed dysbiosis, increasing the abundance of key genera including *Bacteroides*, *Alistipes*, and *Rikenella*, effectively reversing dysbiosis [81,82]. Moreover, fecal microbiota transplanted from these RES-treated animals alleviated inflammation and enhanced intestinal function in recipient mice [82,83].

Furthermore, resveratrol has been shown to enhance intestinal health under oxidative stress by modulating gut microbiota composition; in db/db mice (oral resveratrol 10 mg/kg/day for 12 weeks), Cai et al. (2020) observed increased microbial richness with enrichment of anti-inflammatory taxa, restored Firmicutes/Bacteroidetes balance, improved tight junction expression and barrier function, reduced systemic endotoxin and cytokines, and transferred protection by fecal microbiota transplantation [83]. In diquat-challenged piglets, RES supplementation decreased the abundance of *Firmicutes*, *Actinobacteria*, *Ruminococcaceae* UCG-005, and Eubacterium coprostanoligenes, while promoting beneficial bacteria such as Clostridium sensu stricto and Lachnospiraceae unclassified [84]. Additionally, RES restored microbial diversity by rebalancing key phyla, including *Bacteroidetes*, *Proteobacteria*, and *Firmicutes*. It increased beneficial genera while suppressing potential pathogens like *Lachnoclostridium*, *Acinetobacter*, and *Serratia*, highlighting its role in counteracting oxidative-stress-induced dysbiosis and maintaining gut homeostasis [84].

Resveratrol may also be an effective strategy as it promotes beneficial bacteria (like *Parabacteroides* and *Listipes*) that enhance short-chain fatty acids’ (SCFAs) production. Indeed, metabolites such as short-chain fatty acids (SCFAs), bile acids, and tryptophan derivatives are essential for maintaining mucosal integrity and regulating immune function [82,83]. In diquat-challenged models, RES also elevated metabolites like indole-3-carbinol, 5-hydroxyindole-3-acetic acid, indole, alpha- and beta-dihydroresveratrol, and uridine [84]. Additionally, certain resveratrol-derived microbial metabolites (e.g., 3-(4-hydroxyphenyl)-propionic acid) exhibit anti-inflammatory effects and reinforce the intestinal barrier, partly through the AMPK-SIRT1/NF-κB pathway [82,85]. A summary of primary experimental evidence assessing the impact of resveratrol on gut microbiota is provided in Table 2.

**Figure 2:**
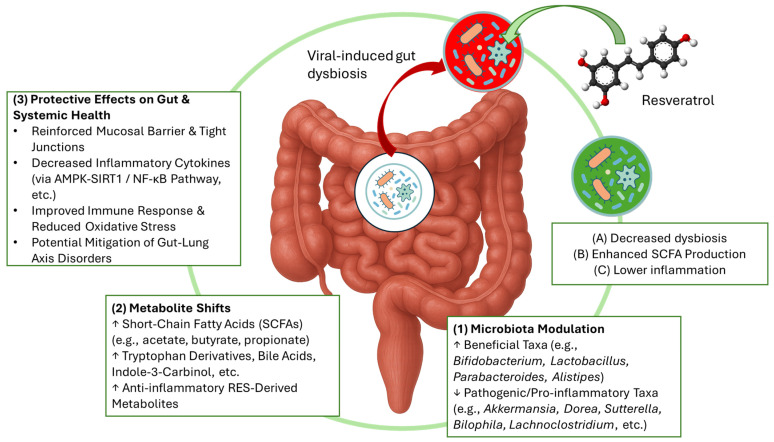
Resveratrol activities in gut microbiota regulations. RES modulates microbiota composition by increasing beneficial taxa such as *Bifidobacterium*, *Lactobacillus*, *Parabacteroides* and *Alistipes*, while decreasing pathogenic and pro-inflammatory taxa including *Akkermansia*, *Dorea*, *Sutterella*, *Bilophila*, and *Lachnoclostridium*. These microbial changes result in metabolite shifts, with enhanced production of short-chain fatty acids (SCFAs), tryptophan derivatives, bile acids, indole-3-carbinol, and anti-inflammatory metabolites derived from RES. Collectively, these effects contribute to decreased dysbiosis, enhanced SCFA production, reduced inflammation, reinforcement of the mucosal barrier and tight junctions, decreased inflammatory cytokines via AMPK-SIRT1/NF-κB pathways, improved immune response, reduced oxidative stress, and potential mitigation of gut–lung axis disorders. In the figure, the “↑” mean increase; and “↓” mean decrease.

Resveratrol, in the end, is capable of inhibiting the anti-inflammatory activity in intestinal cells, via the blockage of pro-inflammatory cytokines synthesis, like COX-2 [86]. Based on the available evidence, we believe that resveratrol supplementation may be useful for children suffering from gut dysbiosis associated with viral infections. Preclinical and limited clinical evidence indicates that resveratrol can modulate the intestinal microbiome and dampen local inflammatory responses, changes that are plausibly linked to the restoration of mucosal homeostasis (Figure 2). However, these data do not support routine supplementation in children at present; rather, resveratrol should be considered a candidate for clinical development and systematic evaluation in age-stratified safety and efficacy trials with integrated microbiome and virological endpoints. 

## 5. Antiviral Activity of Resveratrol in Children

**Figure 3:**
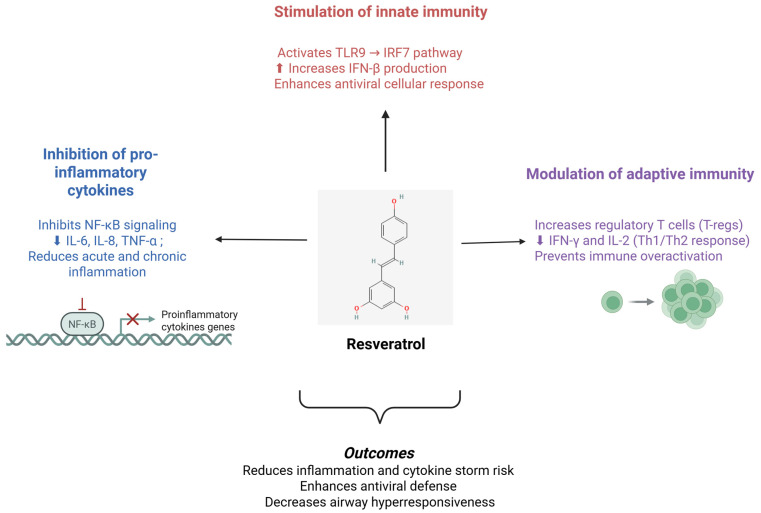
Resveratrol as a modulator of host immune responses. Resveratrol modulates both innate and adaptive immune pathways by activating the TLR9–IRF7 axis and Nrf2/SIRT1 signaling, leading to increased IFN-β and antioxidant responses, while inhibiting NF-κB-mediated cytokine production (IL-6, IL-8, TNF-α). In adaptive immunity, it promotes regulatory T cells and reduces Th1/Th2 cytokines such as IL-2 and IFN-γ, collectively enhancing antiviral defense and reducing excessive inflammation. In the figure, the “↑” mean increase; and “↓” mean decrease.

A summary of the main immunomodulatory pathways through which resveratrol influences the host immune response is presented in Figure 3.

### 5.1. Resveratrol Activity Against SARS-CoV2 and MERS-CoV

The biological activities of Resveratrol suggest its usefulness in COVID-19, such as its powerful antioxidant and anti-inflammatory activities, its inhibition of platelet aggregation, its immunomodulatory effect on immune cells, and its antiviral and antibacterial activities. First of all, resveratrol showed antiviral activity both in adult and child patients, through the inhibition of replication and inflammation induced by respiratory viruses like influenza virus, human coronavirus (HCoV) and human rhinovirus (HRV) [27].

Recent in vitro studies have demonstrated that SARS-CoV and MERS-CoV exhibit sensitivity to resveratrol. The inhibition of MERS-CoV occurs in a dose-dependent manner, preventing MERS-CoV replication and reducing MERS-induced apoptosis. This may be related to the capacity of resveratrol of reducing nucleocapsid (N) protein expression, which is fundamental for CoV replication [87].

Regarding SARS-CoV, synthesized derivatives of resveratrol have been found to inhibit its replication while also mitigating its cytopathic effects [31]. Additionally, molecular docking analyses have indicated that resveratrol establishes a strong interaction with the SARS-CoV-2 spike protein and the human ACE2 receptor complex [88,89]. Since the ACE2 receptor is fundamental for SARS-CoV-2 entry, targeting this enzyme could be favorable. Resveratrol is the most stable compound of the stilbene family in terms of its inhibition of the ACE2 receptor, and it also prevents S1:ACE2 complex formation and the entry of the virus into host cells [88]. It is important that the modulation of ACE2 expression, also present in the GI tract, can interfere with the GI symptoms of COVID-19, such as diarrhea [89]. Based on the regulation of ACE2 expression, dietary intake of resveratrol could prevent or reduce the severity of COVID-19 [90].

Oral administration of resveratrol reduces IL-1B and TNF levels, induces Nrf2 target genes, boosts glutathione synthesis, and shields alveolar epithelial cells from oxidative stress [91]. Resveratrol demonstrates a significant reduction in activity through inhibition NF-κB and IRF-3 binding to endogenous gene promoters, thereby modulating pro-inflammatory cytokine expression (IL-8, IL-1α, IL-6, TNF-α, etc.) [92]. Moreover, resveratrol can activate Nrf2, by downregulating KEAP1 and enhancing SIRT1 deacetylase, and Nf2 is responsible for cell adaptation to oxidative stress and inflammation [93]. Therefore, resveratrol, as a Nrf2-inducing agent, could be utilized to prevent or moderate cytokine storm in COVID-19, especially in association with other anti-inflammatory therapies, i.e., N-acetylcysteine. Consequently, this therapy could reduce cell damage, and it could be useful to prevent respiratory failure and ARDS [94]. Finally, resveratrol has been demonstrated to be an adjunctive antiviral agent to consider, particularly when used at safe supplemental doses [95].

### 5.2. Resveratrol Activity Against Respiratory Syncytial Virus

Respiratory syncytial virus (RSV) is the leading cause of bronchiolitis, one of the main viral infections of the lower respiratory tract in children [96]. It may require hospitalization and cause long-term respiratory sequelae, including asthma and airway hyperresponsiveness (AHR), even 30 years after the first infection [97,98]. RSV is an enveloped virus of the Orthopneumovirus genus (Pneumoviridae family) with a single-stranded, negative-sense RNA genome encoding nine structural proteins [99]. Among these, the matrix (M) protein of the RSV plays a crucial role in facilitating interactions with the host cell’s cytoskeletal components and orchestrating the viral particle’s assembly and release following replication [100]. The impact of this protein on host gene expression, particularly in regulating nuclear genes encoding mitochondrial components, is highly dependent on its chromatin association; in fact, mutations disrupting this interaction significantly impair RSV’s ability to generate infectious virions [101].

In a recent studies, resveratrol has demonstrated potent antiviral activity against RSV by targeting the M protein; spectroscopic and computational analyses revealed that resveratrol binds the M protein, fluorescence quenching experiments indicated a strong interaction, finally, molecular dynamics simulations confirmed the stability of this binding, suggesting that resveratrol interferes with RSV assembly and release, highlighting its potential as a therapeutic agent in pediatric infections due to RSV [102]. The direct antiviral activity of resveratrol also manifests itself by targeting heparan sulfate proteoglycans, rather than interacting with RSV surface proteins like the fusion (F) protein and glycoprotein (G), disrupting the early stages of RSV infection [103].

Experimental research has highlighted resveratrol as a potential antiviral compound, demonstrating its ability to suppress RSV replication while mitigating virus-induced airway inflammation and AHR [104]. This effect appears to be mediated through modulation of host–cell signaling pathways linked to chronic inflammation and lung damage. Specifically, resveratrol was shown to downregulate the virus-induced expression of TRIF (TIR-domain-containing adapter inducing interferon-β) and TBK1 (TANK-binding kinase 1), leading to a reduction in IL-6 levels—a cytokine closely tied to disease severity—as well as decreased INFγ production via the SARM (Sterile α and HEAT/Armadillo motif–containing protein) pathway [105,106].

In vivo studies further confirmed that resveratrol administration resulted in lower viral loads, reduced IFNγ levels, and a diminished presence of inflammatory cells (NK cells, macrophages, and CD3+ T cells) in lung tissues, effectively alleviating airway inflammation and hyperreactivity [107,108]. Notably, resveratrol treatment also led to a decline in neurotrophins, such as the nerve growth factor (NGF) and brain-derived neurotrophic factor (BDNF), which play a critical role in sustaining inflammation associated with RSV infection [101,109].

This dual activity (direct antiviral and anti-inflammatory activity) of resveratrol has been combined in the development of resveratrol nanoparticles (Res NPs), thought to be ingested through nebulized inhalation, offering a new promising formulation strategy for the treatment of RSV-induced pneumonia in children [110].

Despite the promising results of nirsevimab as passive immunoprophylaxis against RSV, no antiviral drugs have been established for RSV treatment to date [111]. In this context, resveratrol has emerged as a potential antiviral candidate due to its ability to inhibit RSV replication and modulate virus-induced inflammatory responses. Anti-RSV mechanisms of action of resveratrol are summarized in Table 3.

### 5.3. Resveratrol Activity Against Other Respiratory Tract Infections in Children

Influenza infections in children remain a major global health concern [112]. In this context, resveratrol’s ability to inhibit both human influenza B and swine influenza A viruses highlights its potential as a valuable antiviral strategy [30,113]. Resveratrol effectively suppressed influenza virus replication in a dose-dependent manner (10–20 µg/mL) by reducing late viral protein translation and preventing the nuclear-to-cytoplasmic transport of viral RNPs, a crucial step before virion assembly and release. These antiviral effects were linked to the inhibition of intracellular signaling pathways such as protein kinase C (PKC) and MAPK [30]. Subsequently, it was shown that a resveratrol analog, in doses ranging from 5 to 20 µg/mL, counteracted virus-induced GSH depletion, restoring cellular redox balance and impairing hemagglutinin maturation [114]. Beyond its direct ability to inhibit viral replication (IC50: 24.7 µM; 50% growth inhibition: >100 µM; therapeutic index: 4), resveratrol has also been shown to modulate the host immune response against various clinical strains of H1N1 and H3N2 influenza A viruses [115,116]. Notably, resveratrol treatment led to increased IFNβ gene expression via the TLR9/IRF7 pathway, suggesting a synergistic antiviral effect between IFNβ and resveratrol in suppressing viral replication) [27,115]. This immunomodulatory activity further supports the role of resveratrol in counteracting influenza infections, complementing its previously reported effects on viral protein translation, RNP translocation, and redox balance restoration.

Despite its promising antiviral activity, the most important limit of resveratrol is represented by its poor oral bioavailability [116]. Building on the demonstrated postulate that resveratrol exhibits dose-dependent antiviral activity in nasal epithelial cell cultures by blocking viral replication—specifically against respiratory viruses such as human rhinovirus, the primary cause of the common cold, which is nearly ubiquitous among the pediatric population during colder months—its potential intranasal application has been proposed for both preventive and therapeutic purposes [117,118]. Resveratrol, also, contrasts the production of IL-6 and IL-8 induced by rhinovirus in nasal epithelia, and the expression of the receptor for HRV on cells, ICAM-1 [117]. Findings from one open-label real-world, randomized study suggest that aerosolized resveratrol combined with carboxymethyl-β-glucan administered via nasal irrigation, following a standard anti-infective and anti-inflammatory treatment, may play a significant role in reducing the recurrence of respiratory infections in pediatric patients [119]. Compared to saline solution, this formulation led to a marked and sustained decrease in nasal obstruction, rhinorrhea, sneezing, coughing, and fever episodes, alongside a reduction in medication use, medical visits, and school absences over a 90-day follow-up period. These results highlight its potential as a supportive therapeutic option for children with recurrent respiratory infections, possibly enhancing mucosal immune defense and prolonging the benefits of conventional treatment [119]. After this study, in line with previous findings, a randomized, double-blind, placebo-controlled trial investigated the effects of intranasal administration of a resveratrol/carboxymethyl-β-glucan solution in infants with the common cold. Treatment with the active compound led to a significant reduction in sneezing and coughing episodes after seven days, particularly in infants infected with HRV [120]. Moreover, an upregulation of TLR-2 expression in children with HRV who were receiving resveratrol/β-glucan suggested a potential enhancement of innate immune defense mechanisms [120]. While these preliminary findings support the role of this formulation in alleviating specific symptoms and reducing respiratory relapses, larger-scale studies are needed to further establish its clinical efficacy and immunomodulatory effects in early childhood [118]. Additionally, a recent prospective single-blind study demonstrated that nasal solutions incorporating resveratrol and carboxymethyl-β-glucan may contribute to reducing the occurrence and severity of wheezing episodes, hospital visits, and oral corticosteroid use in non-atopic preschool children with recurrent upper respiratory tract infections (URTIs), highlighting its potential role as an adjunct therapy in respiratory infection management [121].

Currently, the only published clinical trials examining exclusively the pediatric applications of resveratrol involve its administration via intranasal nebulization (Table 4)**.** As noted, this intranasal route has demonstrated efficacy and may offer a viable strategy to circumvent the compound’s limited oral bioavailability.

### 5.4. Resveratrol Activity Against Rotavirus

The importance of the antiviral activity of resveratrol against rotavirus lies in the fact that rotavirus represents the primary cause of severe acute gastroenteritis in children, associated with a number of deaths annually of 200,000 to date, without availability of an approved and effective antiviral drug [122].

Resveratrol has recently been shown to exhibit notable anti-rotavirus effects both in cell culture and in a neonatal mouse model, underscoring its potential for addressing pediatric rotavirus infections [123]. In vitro experiments using Caco-2 intestinal cells demonstrated that resveratrol at 20 μM effectively inhibited rotavirus replication by reducing viral genomic RNA synthesis, blocking structural protein (VP6) expression, and suppressing virion production. [124,125,126]. Mechanistic studies revealed two principal modes of action. First, resveratrol downregulated heat shock protein 90 (HSP90), a host chaperone critical for multiple stages of the rotavirus life cycle [127]. Second, it curtailed MEK/ERK pathway activation, thereby interrupting a signaling cascade that facilitates viral replication [128,129].

Subsequent in vivo analysis utilized a neonatal mouse model, where oral doses of resveratrol at 10 mg/kg or 20 mg/kg markedly lessened diarrheal severity, diminished viral antigen levels in the gut, and alleviated associated weight loss and inflammatory responses [123,130]. Notably, resveratrol-treated mice exhibited a significantly lower mRNA expression of pro-inflammatory cytokines and chemokines, including IL-2, IL-10, TNF-α, IFN-γ, MIP-1α, and MCP-1, underscoring the compound’s immunomodulatory role [123]. These findings support its promise as an adjunctive or stand-alone intervention for pediatric rotavirus infections, particularly by targeting key host factors (HSP90) and signaling pathways (MEK/ERK) essential to rotavirus replication; however, concerns remain about resveratrol’s oral bioavailability.

### 5.5. Resveratrol Activity Against Norovirus

Our interest moved to resveratrol activity against norovirus, because it represents the principal etiologic agent of sporadic and epidemic gastroenteritis worldwide [15]. With regard to this topic we found only one recent in vitro study in which RAW264.7 murine cells were exposed to murine norovirus (MNV-1) (a commonly used surrogate for human norovirus) and to resveratrol at different dosages at different times (pre-infection 21.32–24.97 µg/mL and after infection at 5.496 µg/mL) [131]. The results indicated that pre-treatment and co-incubation significantly reduced viral titers, implying that resveratrol interferes with virus adsorption or penetration. In contrast, adding resveratrol after infection did not lower MNV-1 replication, suggesting the compound’s main effect occurs before or during early viral entry [131]. Transmission electron microscopy revealed that resveratrol-treated MNV-1 particles were enlarged, suggesting direct alteration of the viral capsid structure. Molecular docking simulations supported this observation by showing strong binding affinity between resveratrol and the MNV-1 major capsid protein (VP1). This interaction likely impairs the virus’s capacity to attach to or fuse with host cells. Additionally, resveratrol regulated the expression of multiple cytokines in infected RAW264.7 cells: it enhanced the antiviral genes TNF-α and Mx while suppressing pro-inflammatory mediators such as IL-6 and IL-1β. Hence, resveratrol appears to block early steps of infection via capsid disruption and to bolster the host immune response against norovirus [131]. Overall, these findings highlight resveratrol as a promising natural agent for controlling norovirus in foods or related settings. Its relatively low toxicity, effective viral inhibition in cell-based models, and synergy with host immune defenses suggest its practical applications as a nutritional or protective additive. Further validation in more complex systems or clinical contexts would help clarify its potential for preventing norovirus outbreaks in humans and especially in children.

## 6. Conclusions

Pediatric viral infections remain a formidable challenge worldwide, not only because of the direct pathogenic effects on the respiratory and gastrointestinal tracts but also due to their profound impact on the gut microbiome. As highlighted throughout this review, a dysregulated intestinal microbiota can exacerbate disease severity and predispose children to secondary complications. Emerging evidence supports resveratrol’s capacity to counteract a broad spectrum of viruses, including those causing respiratory and gastrointestinal diseases in children, such as RSV, influenza virus, rotavirus, and norovirus, by targeting both viral proteins (e.g., RSV M protein and rotavirus VP6) and host factors (e.g., HSP90 and MEK/ERK pathways). Resveratrol appears to exert its antiviral benefits not only by directly targeting viral components but also by reshaping the intestinal microbiome in ways that counter infection-induced dysbiosis. In particular, resveratrol has been shown to reduce the abundance of opportunistic pathogens (e.g., Enterobacteriaceae) and promote beneficial genera such as Bifidobacterium and Lactobacillus, effectively restoring a more balanced microbial community. This microbiome shift correlates with the enhanced production of short-chain fatty acids, improved gut barrier integrity, and dampened local inflammation, factors that collectively strengthen the host’s immunological capacity against viral challenges. By lowering the levels of pro-inflammatory taxa and increasing those that favor anti-inflammatory metabolites, resveratrol helps preserve mucosal homeostasis. In this way, resveratrol’s gut microbiota-modifying properties likely act in tandem with its direct antiviral effects, yielding a two-pronged mechanism that not only impedes viral replication but also fosters a more resilient intestinal environment. Such dual functionality may be particularly valuable in pediatric settings, where viral infections often lead to pronounced dysbiosis and heightened susceptibility to secondary complications. Recent trials focusing on intranasal resveratrol administration have yielded promising clinical outcomes in children with recurrent respiratory infections, notably reducing symptom severity and the frequency of hospital visits. This intranasal route could bypass the compound’s poor oral bioavailability, an issue that has long hampered its clinical application. Despite these encouraging observations, the translation of resveratrol’s laboratory efficacy into clinical settings requires more rigorous trials. Notably, the available pediatric clinical evidence is limited to intranasal formulations; oral use in children remains poorly characterized and requires formal pharmacokinetic and safety assessment. First, larger, multicenter clinical trials are needed to confirm efficacy and safety of resveratrol in diverse pediatric populations. Second, the optimal dosing and formulation—potentially including nanoparticle-based delivery—should be rigorously investigated to enhance its pharmacokinetics. Third, the specific contributions of resveratrol-induced microbiota shifts in attenuating viral infection need more detailed mechanistic studies, possibly employing advanced multi-omics approaches. Finally, exploring synergistic regimens that combine resveratrol with probiotics or existing antiviral drugs may further improve clinical outcomes by simultaneously targeting viral replication and enhancing gut microbial resilience. Taken together, these considerations underscore resveratrol′s potential as a candidate immunomodulatory agent with possible applicability to pediatric populations, pending formal safety, pharmacokinetic and efficacy evaluation in well-designed clinical trials. Although resveratrol has demonstrated antiviral and immunomodulatory activity across several pediatric viral pathogens, including RSV, influenza, rotavirus, and norovirus, these effects are virus-specific and primarily supported by preclinical models. Therefore, the available evidence does not support its designation as a broad-spectrum or universal antiviral compound. Notably, other stilbenoids such as pinosylvin display comparable antioxidant and anti-inflammatory properties; however, unlike resveratrol, pinosylvin currently lacks robust preclinical or clinical evidence for direct antiviral activity and for modulation of the gut microbiota, which strengthens the translational rationale to prioritize resveratrol for pediatric development [132]. Therefore, we propose a staged development pathway: early phase I/II pediatric studies to establish safety, tolerability, and pharmacokinetics (including metabolite profiling and age-dependent dosing), followed by randomized, placebo-controlled efficacy trials that include clinical, virological, and microbiome functional endpoints. Concurrent mechanistic studies using multi-omics and barrier integrity/readout assays (e.g., short-chain fatty acids, zonulin, or fecal calprotectin) will be necessary to clarify the extent to which microbiota shifts mediate clinical benefit. In summary, resveratrol presents a biologically plausible, dual-mechanism approach, with direct antiviral activity coupled with microbiome modulation, that merits clinical development. Well-designed pediatric safety and efficacy trials, together with mechanistic multi-omics work, are required to determine whether the preclinical promise can be translated into safe, evidence-based interventions for children.

## Figures and Tables

**Table 1:** Specific gut microbiota alterations due to viral infections in children.

Virus	Gut Microbiota Alterations	Reference
SARS-CoV2	Increased representation of Bacteroidetes and Firmicutes; decreased Proteobacteria. Significant increase in opportunistic/environmental bacteria (*Pseudomonas*, *Herbaspirillum*, *Burkholderia*, *Streptococcus*, *Erysipelatoclostridium*, *Rothia*, *Actinomyces*, and *Veillonella*). Reduction in typical commensals.	[54]
RSV	Enrichment of S24_7, *Clostridiales*, *Odoribacteraceae*, *Lactobacillaceae*, and *Actinomyces*. *Bacteroides*-dominant profile linked to higher likelihood of severe bronchiolitis. Significant beta diversity alterations. Lower alpha diversity observed in severe RSV disease, indicating reduced richness and evenness.	[20,55,56]
Other Respiratory Tract Infections (RRTIs)	Significant reduced alpha diversity; reduction in lactobacilli and bifidobacteria. Reduced *Verrucomicrobia* and *Tenericutes* phyla; increased *Enterococcus* and decreased *Eubacterium.*	[57,58]
Norovirus	Marked dysbiosis, with Veillonella emerging as the dominant genus in infected children, alongside *Enterococcus faecium*. Significant depletion of butyrate-producing genera (*Faecalibacterium*, *Blautia*, *Subdoligranulum*, *Eubacterium hallii group*, *Fusicatenibacter*, *Agathobacter*, *Roseburia*, and *Dorea)*	[59]
Rotavirus	Reduced alpha diversity in RV-infected group vs. controls. Increased Proteobacteria abundance and decreased beneficial microbes.	[60]

**Table 2:** Experimental studies assessing the impact of resveratrol on gut microbiota composition.

Reference	Study Type	Species/Population	Dose/Regimen	Main Findings
[68]	Randomized, placebo-controlled clinical trial (substudy of metabolic trial)	Adult men	Combined polyphenol supplementation arm included resveratrol 80 mg/day	Resveratrol-containing supplementation altered urinary metabolome (microbiota-derived metabolites) and was associated with changes in fecal phyla (decrease in *Bacteroidetes* in men after resveratrol). Evidence indicates resveratrol (within the supplement) modulates host–microbial metabolic output rather than large, reproducible taxonomic shifts in all participants.
[69]	Controlled preclinical study	Rodents	Low-dose dietary resveratrol (reported as low mg·kg^−1^ range, e.g., approx. ~1 mg·kg^−1^·day^−1^ in the low-dose arm).	Resveratrol attenuated colonic inflammation and altered gut community composition; there was a reduction in opportunistic/pathogenic taxa and relative increases in protective genera (*Lactobacillus*, and *Bifidobacterium*) alongside improvements in mucosal damage markers.
[74]	Preclinical study	Human fecal microbiota (in vitro colon simulator)	Continuous administration of a stilbene-based supplement (resveratrol + viniferin and other stilbenes); multiple concentrations/timepoints.	It showed formulation-dependent effects: in some conditions an increase in *Enterobacteriaceae* and a decrease in *Bifidobacteriales* were observed (i.e., not all stilbene mixtures produce the same ‘prebiotic’ outcome); microbial metabolism of stilbenes produced specific metabolites that correlated with shifts in taxa.
[77]	Preclinical study	High-fat diet-fed mice (obesity model)	Resveratrol dietary supplementation (reported, e.g., 200 mg·kg^−1^·day^−1^ given for 8–12 weeks depending on experiment).	Resveratrol reversed high-fat diet-induced dysbiosis, increased relative abundance of Bacteroidetes and selected beneficial genera (e.g., *Bifidobacterium*, *Lactobacillus*) while reducing obesity-associated taxa; it was associated with decreased fat accumulation and improved metabolic endpoints.
[78]	Preclinical study	Human fecal inocula from healthy donors	Anaerobic incubation with several stilbenoids (including resveratrol) at defined concentrations for 0 and 24 h; sequencing at endpoints.	Stilbenoids (resveratrol and others) modulated community composition in donor-dependent fashion; common observations included decreased *Firmicutes/Bacteroidetes* ratio, reductions in certain *Clostridium* spp., and, in several donors, an increase in *Faecalibacterium prausnitzii* (and other beneficial taxa) depending on the stilbenoid. Effects were compound- and donor-specific.
[80]	Randomized, double-blind, placebo-controlled clinical trial	Adult men and women who were overweight/obese (37 completers)	Combined supplement EGCG 282 mg/day + resveratrol 80 mg/day for 12 weeks (epigallocatechin-3-gallate +resveratrol vs. placebo).	In men (not women) epigallocatechin-3-gallate + resveratrol decreased fecal *Bacteroidetes* and tended to decrease *Faecalibacterium prausnitzii*; baseline *Bacteroidetes* abundance predicted the metabolic response to supplementation (increase in fat oxidation). The authors note the contribution of resveratrol per se cannot be isolated because of the combined supplement.
[83]	Preclinical study	Diabetic mice model	Resveratrol administered 10 mg·kg^−1^·day^−1^ by oral gavage for 12 weeks; fecal microbiota transplantation (FMT) from RES-treated donors to recipients was also performed.	Resveratrol increased α-diversity and selectively enriched genera including *Alistipes*, *Odoribacter*, and *Rikenella* (and other taxa associated with improved metabolic/inflammatory profiles); concomitant decrease in taxa linked to endotoxemia.
[84]	Preclinical study	Piglet oxidative-stress model	Dietary resveratrol included in feed. Main reported regimen: resveratrol supplementation at ~90 mg·kg^−1^ feed in challenged piglets (experimental group vs. diquat control). See Methods for timing relative to diquat exposure.	Resveratrol supplementation restored diversity and shifted composition: it decreased Firmicutes (certain Clostridia), Actinobacteria, and specific opportunistic taxa (e.g., *Lachnoclostridium*, *Acinetobacter*, *Serratia*), and increased beneficial taxa such as Clostridium sensu stricto 1 and members of Lachnospiraceae; changes accompanied by altered metabolome.
[85]	Preclinical study	In vitro (intestinal epithelial barrier assays) and complementary in vivo assays assessing barrier function and metabolite effects	Study focused on resveratrol and its microbial metabolites (e.g., 3-(4-hydroxyphenyl)-propionic acid, and dihydroresveratrol). Concentrations in cell assays were in the micromolar range (physiologically relevant); in vivo dosing details are model-dependent—see full text for exact mg·kg^−1^ regimens.	The paper shows that microbial metabolites derived from resveratrol (not only parent molecule) enhance tight junction protein expression and reduce cytokine production; metabolites such as 3-(4-hydroxyphenyl)-propionic acid exert anti-inflammatory effects and contribute to barrier integrity.

**Table 3:** Different anti-RSV mechanisms of resveratrol.

Affected Proteins/Pathways	Mechanism Type	Cellular Response	Reference	Study Type
Heparan sulfate proteoglycans (HSPGs);	Protein–carbohydrate interaction;	Disruption of early stages of infection;	[103]	In vivo animal study
HRSV Matrix Protein	Protein–protein interaction	Inhibition of viral budding	[102]	Cell line study
TLR3, and TRIF signaling pathway	Signaling pathway modulation	Reduction in airway inflammation and hyperresponsiveness	[108]	In vivo animal study

**Table 4:** Clinical trials assessing efficacy and safety of resveratrol in pediatric populations affected by viral infections.

Study Reference	Study Type	Population	Intervention	Outcomes	Key Findings
[121]	Prospective single-blind randomized controlled trial.	A total of 42 non-atopic preschool children (39 completed follow-up); mean age 4.2 years (range 3.6–5.0); 24 males, 15 females, with recurrent wheezing triggered by upper respiratory tract infections.	Nasal solution containing resveratrol (0.05%) and carboxymethyl-β-glucan (0.33%) administered four times daily for 7 days at the onset of upper airway symptoms.	Fewer wheezing episodes, reduced hospital admissions, and decreased oral corticosteroid usage, implying a decrease in infection severity and respiratory complications.	The use of nasal lavage as a delivery method may contribute to the effectiveness of the treatment via direct targeting. The combination of resveratrol and carboxymethyl-β-glucan suggests a potential synergistic effect.
[120]	Randomized double-blind trial.	A total of 89 infants with respiratory infection symptoms.	Nasal resveratrol + carboxymethyl-β-glucan solution, 3 drops/nostril, 4×/day for 7 days.	Marked reduction in sneezing and coughing, especially in infants with HRV; upregulation of TLR-2 expression, suggesting enhanced innate immune defense.	Highlights resveratrol’s ability to alleviate cold symptoms and reduce relapse in early childhood, reinforcing its supportive antiviral effect.
[119]	Open-label, real-world, randomized study.	A total of 82 children (49 males, mean age 8.1 years) with acute rhinopharyngitis and recurrent respiratory infections.	Resveratrol + carboxymethyl-β-glucan nasal irrigation, for 20 days.	Significant decreases in nasal obstruction, rhinorrhea, sneezing, cough, fever episodes, medication usage, medical visits, and school absences over a 90-day follow-up, compared with saline solution.	Indicates resveratrol’s supportive role in managing recurrent pediatric respiratory infections, potentially extending benefits of standard treatments.

## Data Availability

No new data were created or analyzed in this study. Data sharing is not applicable to this article.

## Abbreviations

The following abbreviations are used in this manuscript:

ACE2	Angiotensin-converting enzyme 2
AHR	Airway hyperresponsiveness
ALRI	Acute lower respiratory infection(s)
ARDS	Acute respiratory distress syndrome
BDNF	Brain-derived neurotrophic factor
CC50	Concentration causing 50% cytotoxicity
CCK-8	Cell Counting Kit-8
CPE	Cytopathic effect
COVID-19	Coronavirus disease 2019
db/db	Diabetic (db/db) mice genotype
DMEM	Dulbecco’s Modified Eagle’s Medium
EC50	Half-effective inhibitory concentration
ELISA	Enzyme-linked immunosorbent assay
F/B ratio	Firmicutes/Bacteroidetes ratio
FBS	Fetal bovine serum
GI	Gastrointestinal
GAPDH	Glyceraldehyde 3-phosphate dehydrogenase
HCoV	Human coronavirus
HSP90	Heat shock protein 90
HSPGs	Heparan sulfate proteoglycans
HRV	Human rhinovirus
HT-29	Human colorectal adenocarcinoma cell line (HT-29)
IFN-γ	Interferon-gamma
IFN-β	Interferon-beta
IC50	Half-maximal inhibitory concentration
ICAM-1	Intercellular adhesion molecule 1
IL-1β	Interleukin-1 beta
IL-1α	Interleukin-1 alpha
IL-2	Interleukin-2
IL-6	Interleukin-6
IL-8	Interleukin-8
IL-10	Interleukin-10
KEAP1	Kelch-like ECH-associated protein 1
MAPK	Mitogen-activated protein kinase
MEK	Mitogen-activated protein kinase kinase
MERS-CoV	Middle East respiratory syndrome coronavirus
Mx	Myxovirus resistance protein (Mx)
MNV-1	Murine norovirus 1
MOI	Multiplicity of infection
NGF	Nerve growth factor
NF-κB	Nuclear factor kappa B
Nrf2	Nuclear factor erythroid 2–related factor 2
PFU	Plaque-forming units
PKC	Protein kinase C
PVDF	Polyvinylidene difluoride
qRT-PCR	Quantitative reverse-transcription PCR
RNPs	Ribonucleoproteins
Res NPs	Resveratrol nanoparticles
RNA	Ribonucleic acid
RSV	Respiratory syncytial virus
RRTIs	Recurrent respiratory tract infections
RT-PCR	Reverse-transcription polymerase chain reaction
SCFAs	Short-chain fatty acids
SIRT1	Sirtuin 1
SARM	Sterile α and HEAT/Armadillo motif–containing protein
SDS-PAGE	Sodium dodecyl sulfate–polyacrylamide gel electrophoresis
SARS-CoV-2	Severe acute respiratory syndrome coronavirus 2
TBK1	TANK-binding kinase 1
TLR	Toll-like receptor
TLR2/TLR3/TLR9	Toll-like receptor 2, 3, and 9
TNF-α	Tumor necrosis factor alpha
TRIF	TIR-domain-containing adapter inducing interferon-β
URTIs	Upper respiratory tract infections
VZV	Varicella-zoster virus
Caco-2	Human intestinal epithelial cell line
MA-104	Monkey kidney cell line (MA-104)
RAW264.7	Murine monocyte-macrophage cell line
DMSO	Dimethyl sulfoxide
PBS	Phosphate-buffered saline
PBST	PBS with Tween-20
IFA	Indirect immunofluorescence assay
CCK-8	Cell Counting Kit-8 (viability assay)
